# Schizophrenia and Inflammation Research: A Bibliometric Analysis

**DOI:** 10.3389/fimmu.2022.907851

**Published:** 2022-06-09

**Authors:** He-Li Sun, Wei Bai, Xiao-Hong Li, Huanhuan Huang, Xi-Ling Cui, Teris Cheung, Zhao-Hui Su, Zhen Yuan, Chee H. Ng, Yu-Tao Xiang

**Affiliations:** ^1^ Unit of Psychiatry, Department of Public Health and Medicinal Administration, & Institute of Translational Medicine, Faculty of Health Sciences, University of Macau, Macao, Macao SAR, China; ^2^ Centre for Cognitive and Brain Sciences, University of Macau, Macao, Macao SAR, China; ^3^ Institute of Advanced Studies in Humanities and Social Sciences, University of Macau, Macao, Macao SAR, China; ^4^ The National Clinical Research Center for Mental Disorders & Beijing Key Laboratory of Mental Disorders, Beijing Anding Hospital & the Advanced Innovation Center for Human Brain Protection, Capital Medical University, Beijing, China; ^5^ Department of Nursing, The First Affiliated Hospital of Chongqing Medical University, Chongqing, China; ^6^ Department of Business Administration, Hong Kong Shue Yan University, Hong Kong, Hong Kong SAR, China; ^7^ School of Nursing, Hong Kong Polytechnic University, Hong Kong, Hong Kong SAR, China; ^8^ School of Public Health, Southeast University, Nanjing, China; ^9^ Department of Psychiatry, The Melbourne Clinic and St Vincent’s Hospital, University of Melbourne, Richmond, VIC, Australia

**Keywords:** schizophrenia, inflammation, bibliometrics, VOSviewer, hotspots

## Abstract

**Background:**

Schizophrenia (SCZ) is a severe psychiatric disorder that involves inflammatory processes. The aim of this study was to explore the field of inflammation-related research in SCZ from a bibliometric perspective.

**Methods:**

Regular and review articles on SCZ- and inflammation-related research were obtained from the Web of Science Core Collection (WOSCC) database from its inception to February 19, 2022. R package “bibliometrix” was used to summarize the main findings, count the occurrences of the top keywords, visualize the collaboration network between countries, and generate a three-field plot. VOSviewer software was applied to conduct both co-authorship and co-occurrence analyses. CiteSpace was used to identify the top references and keywords with the strongest citation burst.

**Results:**

A total of 3,596 publications on SCZ and inflammation were included. Publications were mainly from the USA, China, and Germany. The highest number of publications was found in a list of relevant journals. Apart from “schizophrenia” and “inflammatory”, the terms “bipolar disorder,” “brain,” and “meta-analysis” were also the most frequently used keywords.

**Conclusions:**

This bibliometric study mapped out a fundamental knowledge structure consisting of countries, institutions, authors, journals, and articles in the research field of SCZ and inflammation over the past 30 years. The results provide a comprehensive perspective about the wider landscape of this research area.

## 1 Introduction

Schizophrenia (SCZ), a severe psychiatric disorder with strong heritability, is characterized by persistent delusions and hallucinations (1). Approximately 24 million people worldwide suffer from SCZ, which has an onset from late adolescence to early adulthood (2). Patients with SCZ often have a significantly reduced life expectancy than the general population (3).

In recent years, the role of inflammation in the pathogenesis of SCZ has gained wide attention. Inflammation is an adaptive biological response activated by a poisonous stimulus (4). For instance, one study found increased concentrations of inflammatory cytokines in SCZ patients compared with healthy controls (5). A meta-analysis revealed that patients with SCZ had increased levels of pro-inflammatory cytokine compared to healthy control subjects (6). A longitudinal study also found that a higher serum C-reactive protein level at the age of 15 or 16 years was associated with a greater risk of SCZ in adulthood (7). As such, certain inflammatory biomarkers could serve as possible treatment targets in SCZ. A meta-analysis of 26 randomized controlled trials revealed that anti-inflammatory agents such as estrogens and aspirin may have therapeutic potential for SCZ (8). Despite the rapid growth of published literature on the topic of inflammation and SCZ, accurate and useful information such as the number of relevant publications, countries, institutions, journals, authors, and the frequently used keywords in inflammation-related research in SCZ remains lacking.

Bibliometrics is a widely used approach for examining academic publications (9). With the emergence of scientific databases such as Web of Science (WOS), research data is currently easily accessible, which facilitates the development of bibliometric research (10). Bibliometrics is a comprehensive method comprising quantitative and qualitative analyses that can reveal various features of publications, such as identifying countries, journals, authors, and institutions contributing to a research area, showing commonly cited studies and frequently used keywords, and establishing the cooperation between countries, institutions, and authors in a specific scientific research field (11). Moreover, the bibliometric method can conveniently provide new researchers with an overview of the evolution and developmental frontiers of a certain research field (12). Several bibliometric analyses have investigated the publication trend on either the field of SCZ or inflammation alone (13–16). For instance, a bibliometric study of 51,117 articles on SCZ published from 1975 to 2020 found that “inflammation” has been a trending keyword in recent years (16). Another bibliometric analysis provided a comprehensive overview of the development of motivation in SCZ (17). To date, however, no bibliometric analysis on the topic of SCZ and inflammation together has been published. To fill this gap, this bibliometric analysis constructed a global map of the scientific publications on SCZ and inflammation related research.

## 2 Methods

### 2.1 Data Acquisition and Search Strategy

Web of Science (WOS) is one of the most commonly used academic database sources which contains more than 12,000 influential journals (18). Compared with other databases such as Scopus and PubMed, it is widely recognized as the most comprehensive and reliable database for bibliometric analysis (18, 19). In this study, the relevant literature was searched and exported in the Web of Science Core Collection database (WOSCC) on February 19, 2022. WOSCC with all editions (i.e., Science Citation Index Expanded (SCI-expanded), Social Sciences Citation Index (SSCI), Conference Proceedings Citation Index-Science (CPCI-S), Emerging Sources Citation Index (ESCI), Conference Proceedings Citation Index – Social Science & Humanities (CPCI-SSH), Arts & Humanities Citation Index (A&HCI), Book Citation Index – Science (BKCI-S), Book Citation Index – Social Sciences & Humanities (BKCI-SSH), Index Chemicus (IC), and Current Chemical Reactions (CCR-EXPANDED)) was used. Following previous studies, the search strategy was set as the following (16): (TS = (inflammatory OR inflammation OR inflammations)) AND TS = (schizophren*)—literature types including regular and review articles, with no limitation in publication language. Relevant articles were exported and stored in the form of plain.txt (including full record and cited references) for further analyses.

### 2.2 Data Analysis

Bibliometric analyses were performed using three tools, namely, R version 3.5.6 (20), VOSviewer (21), and CiteSpace (22).

*Bibliometrix* is an R package containing a series of functions for scientometric quantitative research. In this study, it was used to 1) summarize the number of publications and citations of bibliometric analysis; 2) identify annual cumulative occurrences of the top keywords/terms; 3) calculate the cooperation frequency among countries; and 4) visualize a three-field plot of the Keywords Plus analysis.

VOSviewer is a distance-based bibliometric tool that focuses on the visualization of bibliometric networks (23). It can assign a set of closely related nodes into several clusters, where the same color indicates higher correlations of nodes (24). Additionally, VOSviewer supports the overlay visualization map, in which the color and distance of nodes represent how nodes are distributed in two-dimensional spaces (i.e., the time and associations) (25). VOSviewer was used in this study to perform 1) a co-authorship network that explored the authors’ and their institutions’ collaboration networks and 2) co-occurrence network that reflected the associations between authors’ keyworks.

CiteSpace is a free Java application, with a focus on dynamic visualizations that reflect the evolution of the bibliometric network over time (26). In this study, it was used to identify highly cited references and keywords with the strongest citation burst during a certain period.

Moreover, the generalized additive model was used to estimate the trend and number of publications through R with the *mgcv* package (27). An online bibliometric website (https://bibliometric.com/) was used to visualize the international collaboration between countries.

## 3 Results

### 3.1 Publication Summary

A total of 3,596 publications on SCZ and inflammation were included, with 2,757 regular and 839 review articles. Of these articles, 3,518 (97.8%) were published in English, while 20 were published in French, 20 in German, 12 in Spanish, 12 in Polish, 6 in Japanese, 3 in Turkish, 2 in Russian and 1 each published in Chinese, Hungarian, and Italian languages.

**Figure 1:**
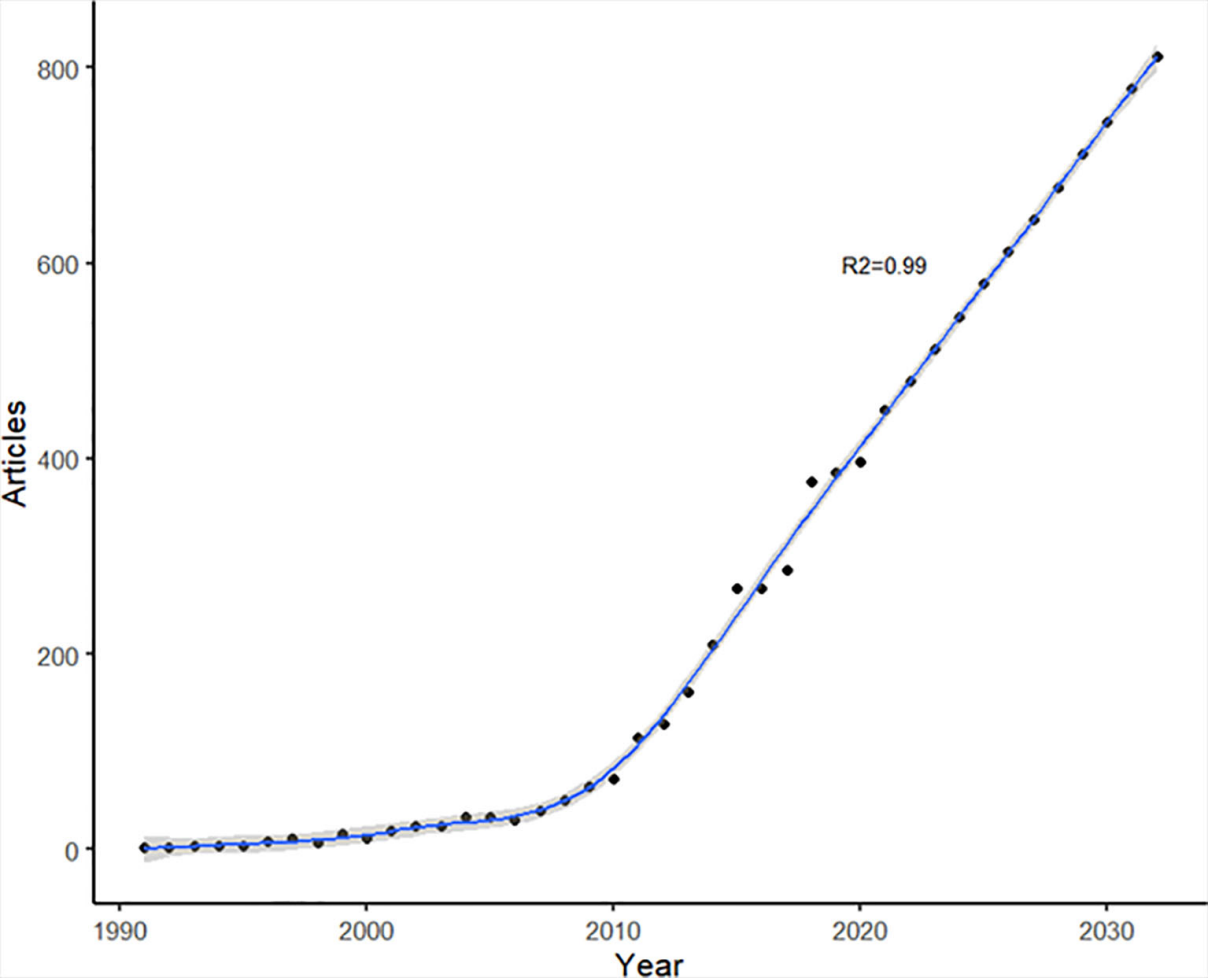
Number of annual articles on schizophrenia and inflammation by years.

**Figure 1** illustrates the number and trend of the annual publications on SCZ and inflammation. The annual growth rate was 10.72%. The first article was published in 1991, with the growth of the number of articles increasing steadily from 2 in 1991 to 72 in 2010. In the following 10 years, the number of articles has exponentially increased, growing from 115 articles in 2011 to 449 in 2021; there was a substantial growth from 286 in 2017 to 371 in 2018 (growth rate: 38.4%). Additionally, a generalized additive model was used to assess the relationship between the number of articles and the publication year (excluding 2022), which showed that the model fitted perfectly with the publications’ annual trend (R^2^ = 0.999). According to the prediction curve, during the next 10 years till 2032, the trend of the literature on SCZ and inflammation will continue to increase, with an expected number of 811 in 2032.

### 3.2 Analysis of the Most Productive Countries

**Figure 2:**
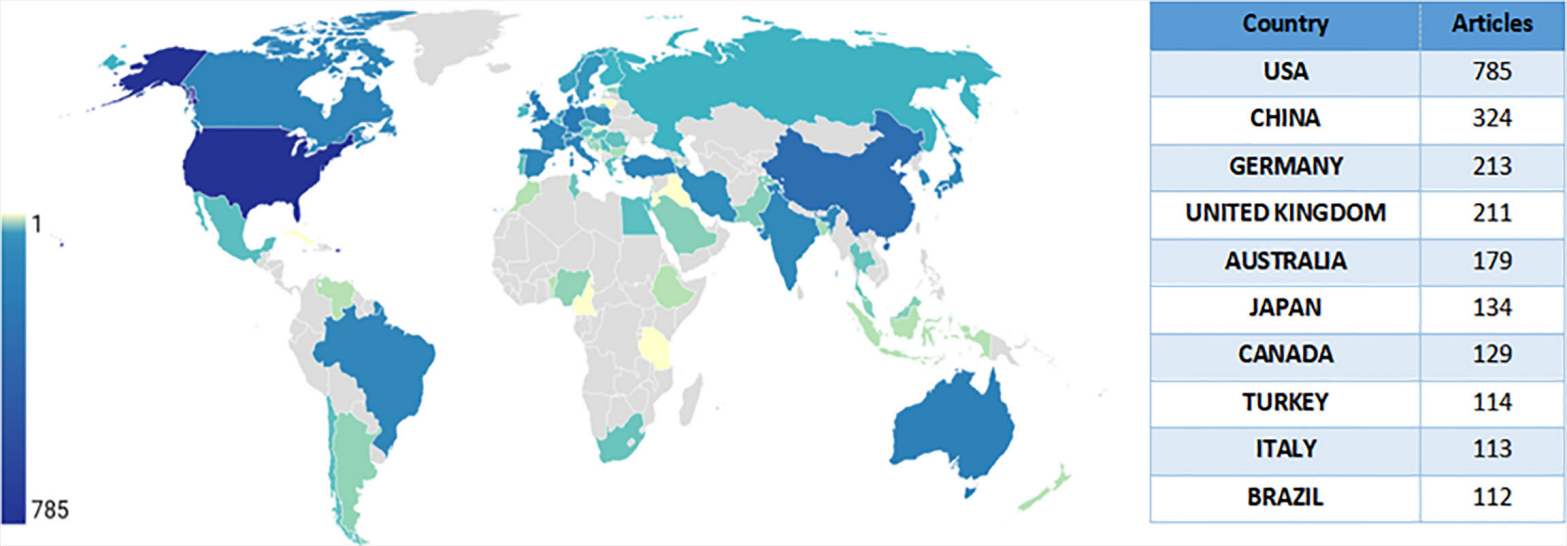
A world map depicting the contribution of each country based on publication counts.

**Figure 2** shows the number of articles in each country/territory. The USA had the greatest number of publications (N = 785), followed by China (N = 324), Germany (N = 213), and UK (N = 211). Each of the remaining countries published less than 200 articles.

**Supplement Figure 1** shows the international cooperation between countries/territories. Most of the research collaborations occurred between North American, European, Oceania, and East Asian countries, with the most frequent cooperation between the USA and China (frequency = 106), followed by the cooperation between the USA and UK (frequency = 101) and between Australia and Thailand (frequency = 74).

### 3.3 Analysis of the Most Productive Institutions

**Figure 3:**
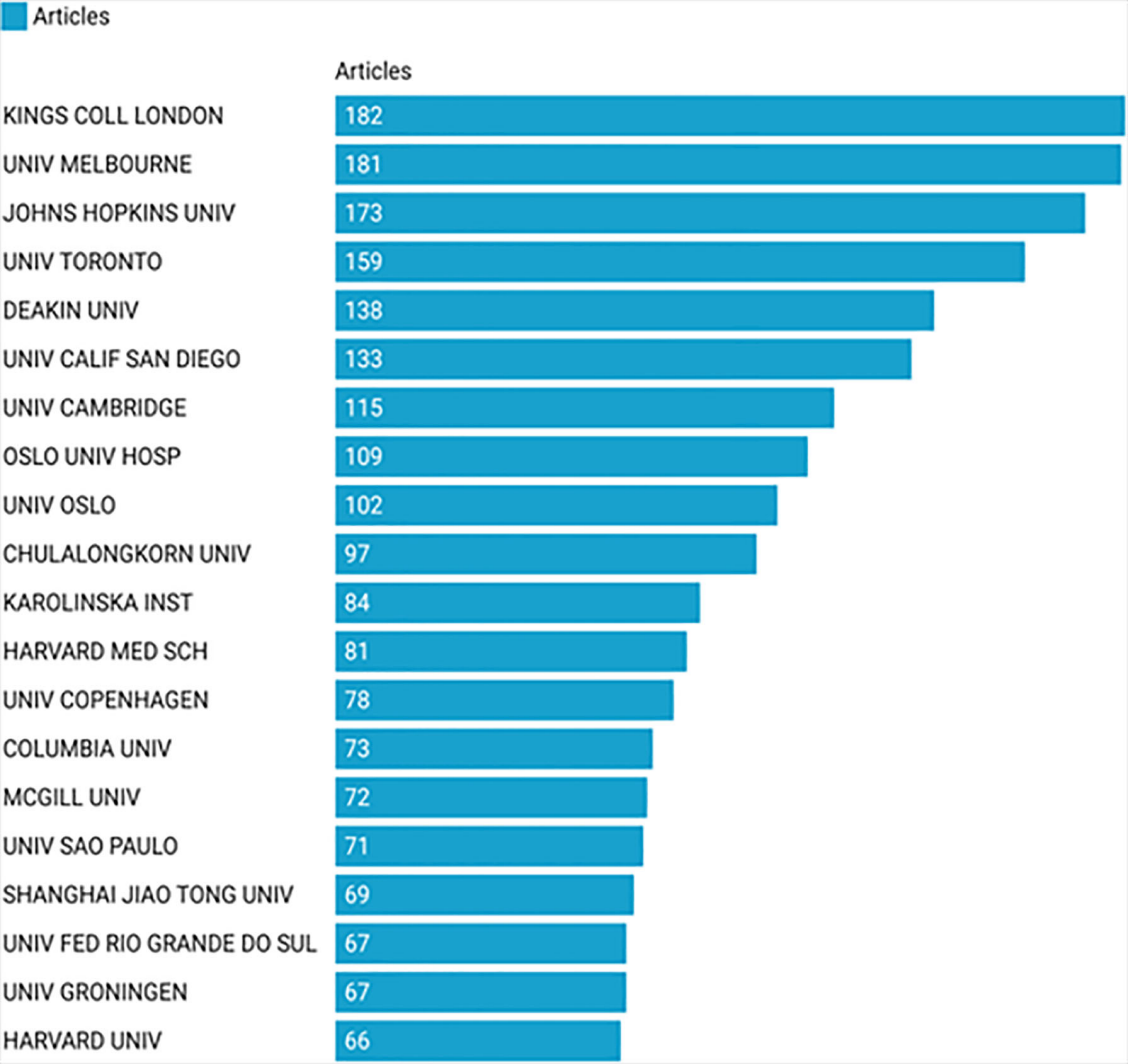
Top 10 institutions with most publications in the field of schizophrenia and inflammation research.

Approximately 3,800 institutions contributed to the research on SCZ and inflammation. **Figure 3** shows the 10 most productive institutions, with the UK, USA, Australia, and Norway each had two most productive institutions in the list. The co-authorship analysis of affiliations could estimate relationships among different institutions by the number of coauthored publications. In the overlay network of co-authorship analysis, the size of the circle indicates the number of publications and the color represents the average commencement year of publications in the specific research field in each institution. As shown in **Supplement Figure 2**, 60 institutions, with the minimum of 20 publications, were identified. Researchers at University of Cambridge in the UK and Johns Hopkins University in the USA started early in the research field of SCZ and inflammation. In contrast, those at Deakin University in Australia and at Chulalongkorn University in Thailand conducted more recent research in this area.

### 3.4 Analysis of the Higher-Impact Journals

The articles on SCZ and inflammation research were published across 918 journals. **Table 1** displays the top 10 journals with the greatest number of publications and their recent impact factor (IF). In terms of Journal Citation Reports (JCR), most of the journals were classified into Q1 (90%), with 70% of the journal classified as psychiatry. As for the publisher location, of the top 10 journals, five out of the top 10 journals were in the USA, 3 in the UK, and 2 in Netherlands.

**Table 1:** Top 10 journals with most publications in the field of schizophrenia and inflammation research.

Ranking	Sources	Articles	Country	IF	JCR-c
1	Schizophrenia Research	165	Netherlands	4.939	Q1
2	Brain Behavior and Immunity	143	USA	7.217	Q1
3	Psychiatry Research	103	Netherlands	3.222	Q1
4	Progress in Neuro-psychopharmacology & Biological Psychiatry	85	UK	5.067	Q1
5	Frontiers in Psychiatry	76	USA	4.157	Q2
6	Translational Psychiatry	73	USA	6.222	Q1
7	Journal of Psychiatric Research	66	UK	4.791	Q1
8	PLOS One	62	USA	3.240	Q1
9	Molecular Psychiatry	54	UK	15.992	Q1
10	Schizophrenia Bulletin	53	USA	9.306	Q1

IF, impact factor (2020–2021); JCR-c, Journal Citation Reports category (2021).

### 3.5 Analysis of the Most Influential Authors

As presented in **Table 2**, 15,692 authors contributed to SCZ- and inflammation-related publications. Michael Maes’ team/lab was the most productive which published 98 articles (h_index = 32), followed by Michael Berk (60 articles with h_index = 27), Marion Leboyer (55 articles with h_index = 21), and Norbert Muller (47 articles with h_index = 28). **Supplement Figure 3** shows the maps of cooperation between researchers; the minimum number of papers per author was set as 10. Of the remaining 94 authors, there were several communities, with each community clustering near one or two frequently published authors. The links between different communities were relatively sparse, which indicates that cooperation between research teams/labs conducting SCZ- and inflammation-related studies was not well established.

**Table 2:** Top 10 authors with the most publications in the field of schizophrenia and inflammation research.

Ranking	Authors	Articles	h_index
1	MAES M Maes, Michael	98	32
2	BERK M Berk, Michael	60	27
3	LEBOYER M Leboyer, Marion	55	21
4	MULLER N Muller, Norbert	47	28
5	YOLKEN RH Yolken, Robert	40	23
6	LEZA JC Leza, Juan C	39	17
7	MEYER U Meyer, Urs A	34	25
8	TEIXEIRA AL Teixeira, Antonio L	34	20
9	WEICKERT CS Weickert, Cynthia Shannon	34	17
10	ANDREASSEN OA Andreassen, Ole A	33	16

### 3.6 Analysis of Research Hotspots

#### 3.6.1 Most Cited Articles

Citation analysis is a valuable method to assess the most highly cited articles; the frequency of citations could reflect the influence of articles in a specific research field (28). **Supplement Table 1** shows the 10 most cited articles, all of which were published between 1997 and 2019, and 80% of them have reached more than 500 citations. Of these articles, the top three were meta-analyses, with two published in Biological Psychiatry. Specifically, the article entitled “Meta-analysis of cytokine alterations in schizophrenia: clinical status and antipsychotic effects” published in 2011 is the top-cited article in this field with 1,031 citations (6). This meta-analysis included 40 studies and revealed that certain cytokines (e.g., IL-1β, IL-6, and TGF-β) were state markers of acute exacerbations, while others (e.g., IL-12, IFN-γ, TNF-α, and sIL-2R) were trait markers. It also suggested that the association between cytokine and acute exacerbations of SCZ was independent of any antipsychotics.

#### 3.6.2 Analysis of References With Citation Burst

**Figure S4** illustrates the top 20 references with the highest citation burst. The minimum duration of the burst was 5 years, while the blue line represents the observed time interval from 1992 to 1991 and the red line represents the burst duration. Of these articles, the article entitled “Inflammatory Cytokine Alterations in Schizophrenia: A Systematic Quantitative Review” published in Biological Psychiatry has the strongest citation burst value during 2008–2013. Moreover, the citation burst for several articles is still ongoing, such as “Postmortem evidence of cerebral inflammation in schizophrenia: a systematic review,” which suggests that such research topics are likely to remain popular in the future and may become potential frontiers in the SCZ and inflammation research field.

#### 3.6.3 Analysis of the Most Frequently Used Keywords

**Figure 4:**
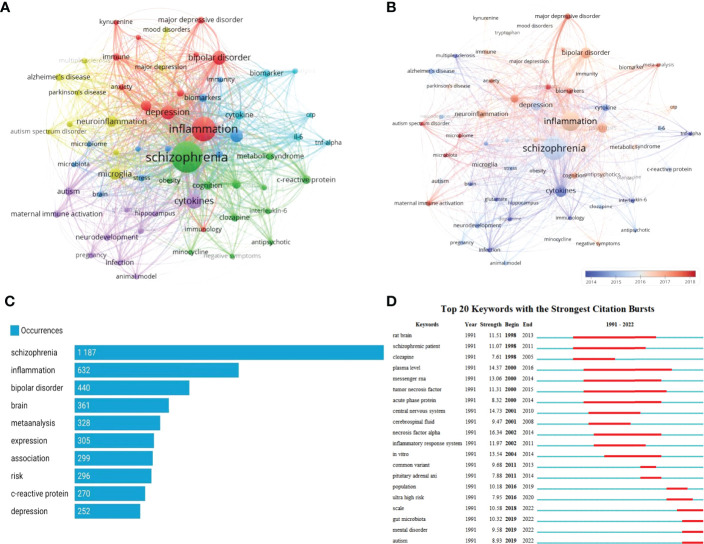
Analysis of the research hotspots on schizophrenia and inflammation (**A**, network visualization of author keywords; **B**, overlay visualization of author keywords; **C**, top 10 most frequent keywords; **D**, visualization map of top 20 keywords with the strongest citations bursts).

Of the 5,906 keywords, with a minimum number of occurrences as 30, 60 keywords met the criteria and were included for analyses. Following a previous study (29), keywords with similar meanings were merged. **Figure 4A** shows the network visualization of these keywords. The size of nodes reflects the occurrence frequency of keywords, while the distance between two nodes reflects the strength of their association. Keywords with a closer distance were classified into the same cluster, which roughly reflects the main topics in the SCZ and inflammation research area. Cluster 1 is colored in red, with the main keywords focusing on psychiatric disorder- and inflammation-related terms such as “bipolar disorder,” “depression,” “immune,” and “immunology,” cluster 2 in green focused on antipsychotics, with the main keywords “clozapine,” “minocycline,” and “olanzapine.” In addition, several terms, such as “c-reactive protein” and “interleukin-6,” were also included in cluster 2. Cluster 3 in blue color focused on the pathways related to SCZ and inflammation, with the main keywords “biomarkers,” “gene system,” and “brain.” Cluster 4 in yellow color focused on the central nervous system (CNS), mainly involving “neuroinflammation,” “microglia,” “Parkinson’s disease,” “Alzheimer’s disease,” and “Multiple sclerosis.” Cluster 5 in purple color focused on “cytokines,” “dopamine,” “glutamate,” and “animal model.” **Figure 4B** presents the overlay visualization of author keywords. The keywords that appeared earlier are colored in blue, while the orange color represents the keywords that have appeared recently. Keywords, such as “cytokines,” “antipsychotic,” “neurodevelopment,” “infection,” and “animal model,” were the major topics in the early period. In contrast, keywords “biomarker,” “immune activation,” “microbiome,” and “neuroinflammation” have been popular topics in recent years.

**Figure 5:**
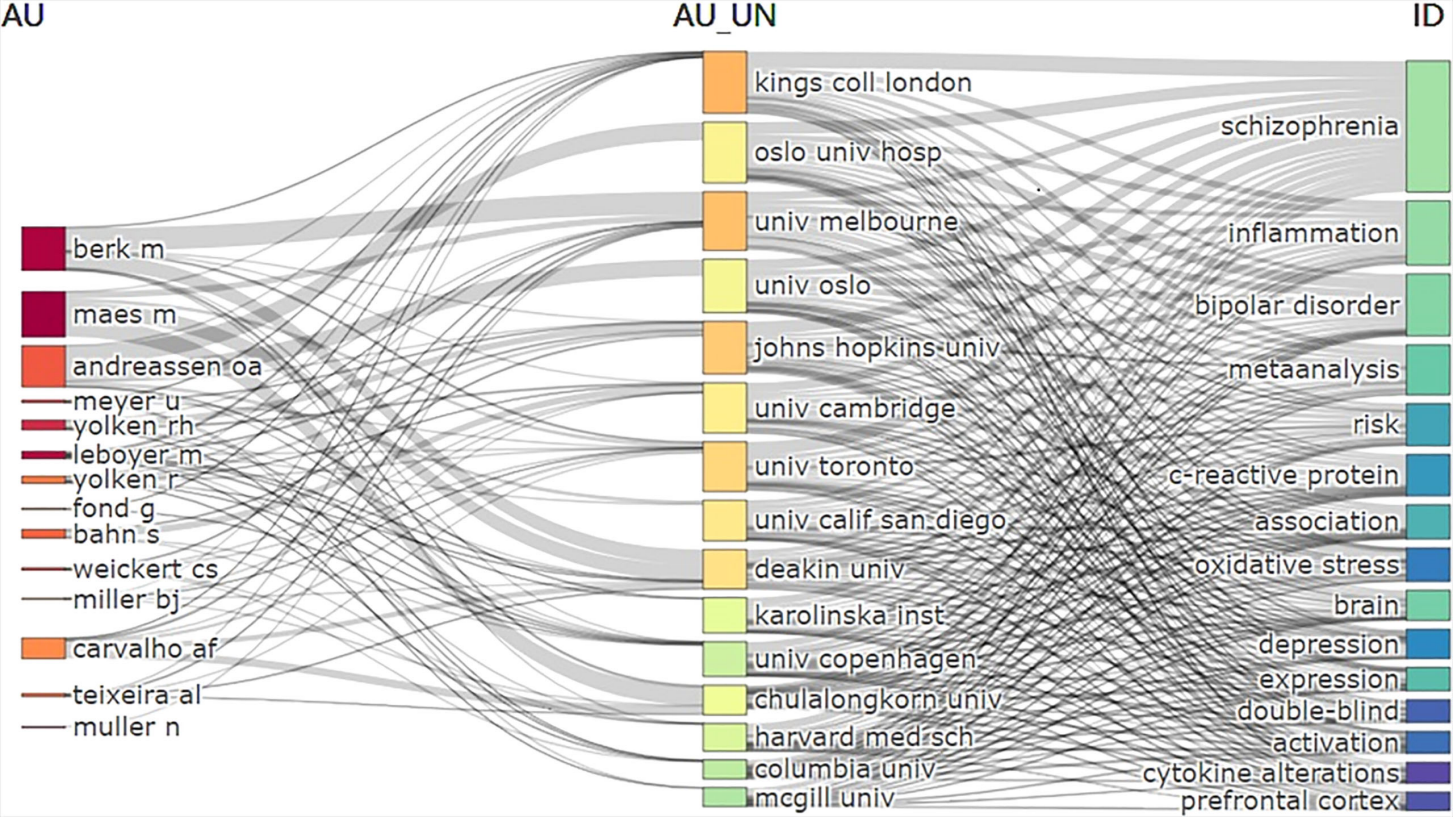
Three-field plot of the Keywords Plus analysis on schizophrenia and inflammation Notes: three-field plot of the keywords plus analysis: (middle field: affiliations; left field: authors; right field: keywords plus).

**Figure 4C** shows the 10 most frequent keywords, with “schizophrenia” being the most used keyword with 1,187 frequencies, followed by “inflammation” (N = 632) and “bipolar disorder” (N = 440). Of the commonly used keywords, “Meta-analysis” (N = 328) was a statistical-related term. **Figure 5** shows the relationships between affiliations, authors, and keywords in the field of SCZ and inflammation research.

#### 3.6.4 Analysis of Keywords With Citation Burst

**Figure 4D** shows the top 20 keywords with the strongest citations bursts, with the minimum duration of the burst being 3 years. The keywords “rat brain” (1998–2013), “plasma level” (2000–2016), “tumor necrosis factor” (2000–2015), and “schizophrenic patients” (1991–2011) have received the longest attention during the past period. The keywords “gut microbiota” (2019–2022), “mental disorder” (2019–2022), and “autism” (2019–2022) have been used more recently, which indicates that these keywords have attracted sufficient attention lately and potentially may be a hot research topic in the future.

## 4 Discussion

This bibliometric analysis study analyzed the development of SCZ- and inflammation-related research in the past 30 years. Articles on SCZ and inflammation showed a growing trend over the past decades, particularly since 2011. A similar trend was observed in a previous bibliometric study on SCZ alone, which found that the publication number has also grown substantially since 2011 (15). The potential reason for the expansion of research might be due to the increasing recognition of the role of inflammation (30) in the development of SCZ (31) and, therefore, increased research funding in this field. More than 90% of the articles were published in English, which is not surprising because the WOSCC database mainly included English journal articles, and English is the most widely used academic language globally (32).

In this bibliometric analysis, most of the relevant articles were published by corresponding authors from several countries such as the USA, China, Germany, and the UK. Similar patterns have been found in bibliometric studies in other fields such as depression (33), bipolar disorders (34), and general neuropharmacology (35). Academic capability is largely determined by the economic status of a country (36). Additionally, governmental expenditure on healthcare may be another important indicator of medical research outputs (37). The USA outspends all other countries in health expenditure with USD10,202 per resident (38), which may partly explain the largest number of publications in the USA. Most of the collaborations on SCZ and inflammation research are also centered on the USA, which is consistent with the prominent contribution of USA in this academic field, which indicates that collaborations between other countries/territories need to be enhanced.

Similar to the findings on the contribution of individual countries on relevant publications, a number of institutions in the UK, USA, and Australia published widely on SCZ and inflammation. In contrast, only one Chinese institution was among the top 15 institutions in the ranking list, even though China is the second most productive country in terms of publications, while one university in Thailand alone published almost 100 articles on SCZ and inflammation research, which is not consistent with the volume of country contribution. Most of these articles were based on collaboration, suggesting that interinstitutional collaboration is an important approach to improving the quantity and quality of publications.

Analyzing the characteristic of international peer-reviewed journals is helpful to understanding the current trends (39, 40). Among the top 10 most active journals in the field of SCZ and inflammation research, most of the publishers are in the USA and Western Europe. Schizophrenia Research was the most productive journal in the SCZ and inflammation research field; not surprisingly, this journal was also the most active journal with the maximum number of articles in other schizophrenia-related academic fields such as motivation in SCZ (17), cognitive behavioral therapy for SCZ (41), and magnetic resonance imaging studies of SCZ (42). In contrast, no publishers are based in East Asia although China and Japan were also two key countries contributing to SCZ and inflammation research. This highlights the importance of developing influential international journals in Asia. The IF of journals is a crucial evaluation indicator. JCR is also a commonly used index for evaluating the quality of journals based on the ranking of IF with four classifications, ranging from the top quartile (Q1) to the fourth quartile (Q4). However, no significant associations were found between number of articles, IF value, and JCR quartile in this study, which indicates that journals may use different approaches to establish research impact. Some journals prefer to publish a large number of articles, while others may prefer high-quality articles that may have more frequent citations with higher IF values or JCR quartile.

The number of articles published by a research team could reflect its activity and contribution in a certain scientific area. In 1995, a team in Thailand published an article on the association of immune-inflammatory variables in SCZ and mania, which has been highly cited (number of citations = 480) (43). The coauthorship between researchers is helpful to exploring existing collaborations and finding potential collaborators. Co-authorship analysis is a useful method to identifying existing partnerships and facilitating developing potential partners. Two teams in Thailand and Australia have collaborated closely, with one of their publications on the pathway between inflammation and bipolar disorder having more than 1,000 citations (44).

Hotspots refers to a scientific topic in a specific research area during a certain period of time, which is one of the key methodologies in bibliometric analysis (29). Citation analysis could reflect the academic influence of publications. Among the 10 most cited articles identified in this study, the key focus was inflammatory cytokine alterations in SCZ patients (45–48). Previous studies found that the imbalance of Th1/Th2 cytokines plays a vital role in SCZ, with a slant toward the Th2 system (45, 49, 50). In addition, IL-6 levels significantly decreased, while sIL-2R, IL-1RA, and IL-6 levels increased in SCZ patients (47). Among the 10 most cited articles, two articles were reviews on the pathology under SCZ and inflammation (51, 52), and another two articles focused on the treatment of psychiatric disorders (53, 54). In contrast, recent research focused more on SCZ- and inflammation-related genetic variants (55, 56). A genome-wide association study found that six immune candidates, namely, DPP*4*, *HSPD1*, *EGR1*, *CLU*, *ESAM*, and *NFATC3*, were associated with schizophrenia (57). Hence, this research field has been focusing on biomarker discovery and the development of precision treatments for SCZ patients. Keyword is an important indicator in scientific research as it reflects the core content of the relevant paper. The co-occurrence analysis of keywords could show the closeness and prevalence of the research topics in scientific areas (58). For the most frequently used keywords, apart from terms “schizophrenia” and “inflammation,” other commonly used keywords focused on the potential mechanisms of SCZ that involve inflammation. Additionally, biomarkers of inflammation c-reactive protein (CRP) appeared frequently. Researchers have found that the association between elevated CRP and SCZ was independent of confounding factors, such as BMI and smoking, which may lead to the development of immune treatments of SCZ (59). Bipolar disorder is another high-frequency keyword; SCZ and bipolar disorder often shared the same clinical attributes (60) and genetic factors (61); thus, they are usually investigated together.

The co-occurrence clustering function roughly divided the whole network into five clusters, with each cluster as a main topic. Cluster 2 in green color included keywords on antipsychotic medications. Some studies found that certain antipsychotic drugs may have immunomodulatory effects through targeting cytokines (62, 63). In addition, other studies found that certain anti-inflammatory drugs (e.g., aspirin) could be used as adjuncts to antipsychotic drugs, and their efficacy and safety in treating schizophrenia might be better than antipsychotics alone (54, 64). Cluster 4 in yellow color contained several CNS-related terms. Neuroinflammation, an innate activation response to an inflammatory stimulus, was often used in connection with microglia (65). Microglia, as the CNS-resident macrophages, has been shown to be involved in the initiation or progression of several CNS disorders, such as Parkinson’s disease, Alzheimer’s disease, multiple sclerosis, and SCZ (66, 67). One explanation is that microglia can facilitate the maturation of neuronal progenitors by secreting insulin-like growth factors; thus, it may play a crucial role in CNS immune responses (68).

The burst detection analysis is an important approach to explore the evolution of research hotspots in an academic interest area. Articles or keywords with high citation bursts imply that they are actively discussed or used during a specific period. Gut microbiota has been an ongoing burst keyword since 2019, which is consistent with the development of microbiota–microglia axis hypothesis in the SCZ and inflammation field. Specifically, gut microbiota work as a regulator of microglial function, and microglia are involved in mediating inflammation and neurodegenerative disorders such as SCZ (69). Compared to healthy controls, immunomodulatory bacterial genera, such as Lactobacilli and Bifidobacteria, are relatively more prevalent in SCZ patients (70). Therefore, targeting a specific gut microbiome may be a future direction in SCZ treatment.

This study has several limitations. First, the data were retrieved from WOSCC alone. Although WOS has been recommended as the most reliable database for bibliometric studies (71–74), some articles may be still missed. Second, the majority of articles were published in English, which may lead to selection bias in terms of publication language. Third, certain inconsistencies may exist in various aspects; for example, one institution may use different names across different periods.

## 5 Conclusion

In conclusion, research on the role of inflammation in SCZ has received growing attention. The substantial growth in the number of annual publications suggests that this research field has gained importance globally, with the USA having the largest number of publications. This study has identified the key researchers and institutions involved in SCZ- and inflammation-related research globally. Schizophrenia Research was the most productive journal in this research field, while Molecular Psychiatry has the highest IF in this field. Inflammatory cytokine and genetic variants have been regarded as the hot topics, while gut microbiota may be a key direction of future research. These findings provide a comprehensive perspective for new researchers and policymakers about the wider landscape of this research field.

## Data Availability Statement

All the data used in the study have been included in the article/**Supplementary Material**.

## Author Contributions

Study design: H-LS, WB, X-HL, CN, Y-TX; data collection, analysis, and interpretation: H-LS, WB, X-HL, HH, X-LC. TC, ZS, ZY; drafting of the manuscript: H-LS, WB, Y-TX; critical revision of the manuscript: CN. All authors contributed to the article and approved the submitted version.

## Funding

The study was supported by the Beijing Municipal Science & Technology Commission (Grant No.: Z181100001718124), Beijing Talents Foundation (Grant No.: 2017000021469G222), and the University of Macau (MYRG2019-00066-FHS).

## Conflict of Interest

The authors declare that the research was conducted in the absence of any commercial or financial relationships that could be construed as a potential conflict of interest.

## Publisher’s Note

All claims expressed in this article are solely those of the authors and do not necessarily represent those of their affiliated organizations, or those of the publisher, the editors and the reviewers. Any product that may be evaluated in this article, or claim that may be made by its manufacturer, is not guaranteed or endorsed by the publisher.
