# Supplementary material for: Schizophrenia and Inflammation Research: A Bibliometric Analysis
Source: Front Immunol. 2022 Jun 9;13:907851. doi: 10.3389/fimmu.2022.907851 (PMC9219580; doi:10.3389/fimmu.2022.907851)
Supplement: Supplementary file 1 [file DataSheet_1.docx]

***Supplementary Material***

**Figure S1. International collaboration analysis and the top 10 collaborations between countries based on schizophrenia and inflammation-related publications**

**Figure S2. Overlay visualization map of worldwide institutions institution co-authorship analysis on schizophrenia and inflammation**

**Figure S3. Network visualization map of author co-authorship analysis on schizophrenia and inflammation**

**Figure S4. Top 20 keywords with the strongest citation bursts on schizophrenia and inflammation**

**Table S1. The top 10 cited articles**

**
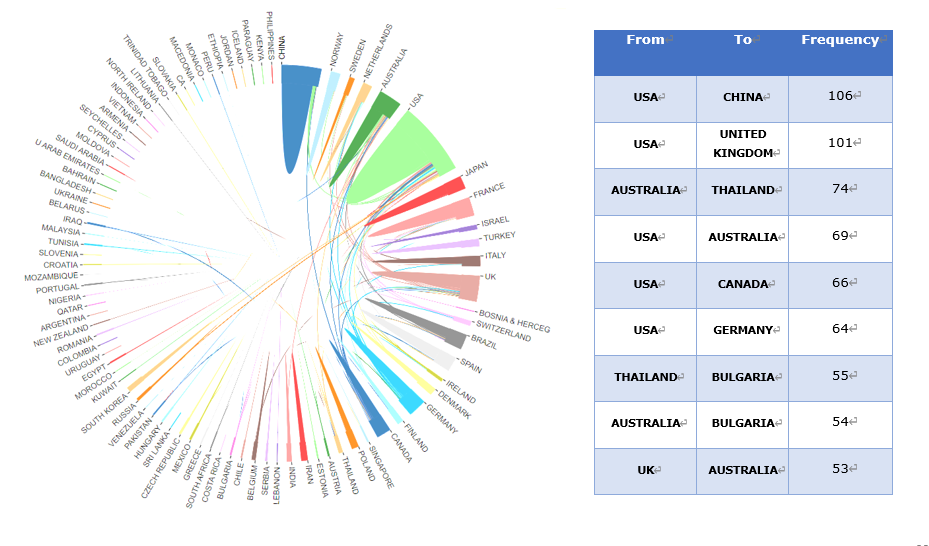
**

**Figure S1.** **International collaboration analysis and the top 10 collaborations between countries based on schizophrenia and inflammation-related publications**


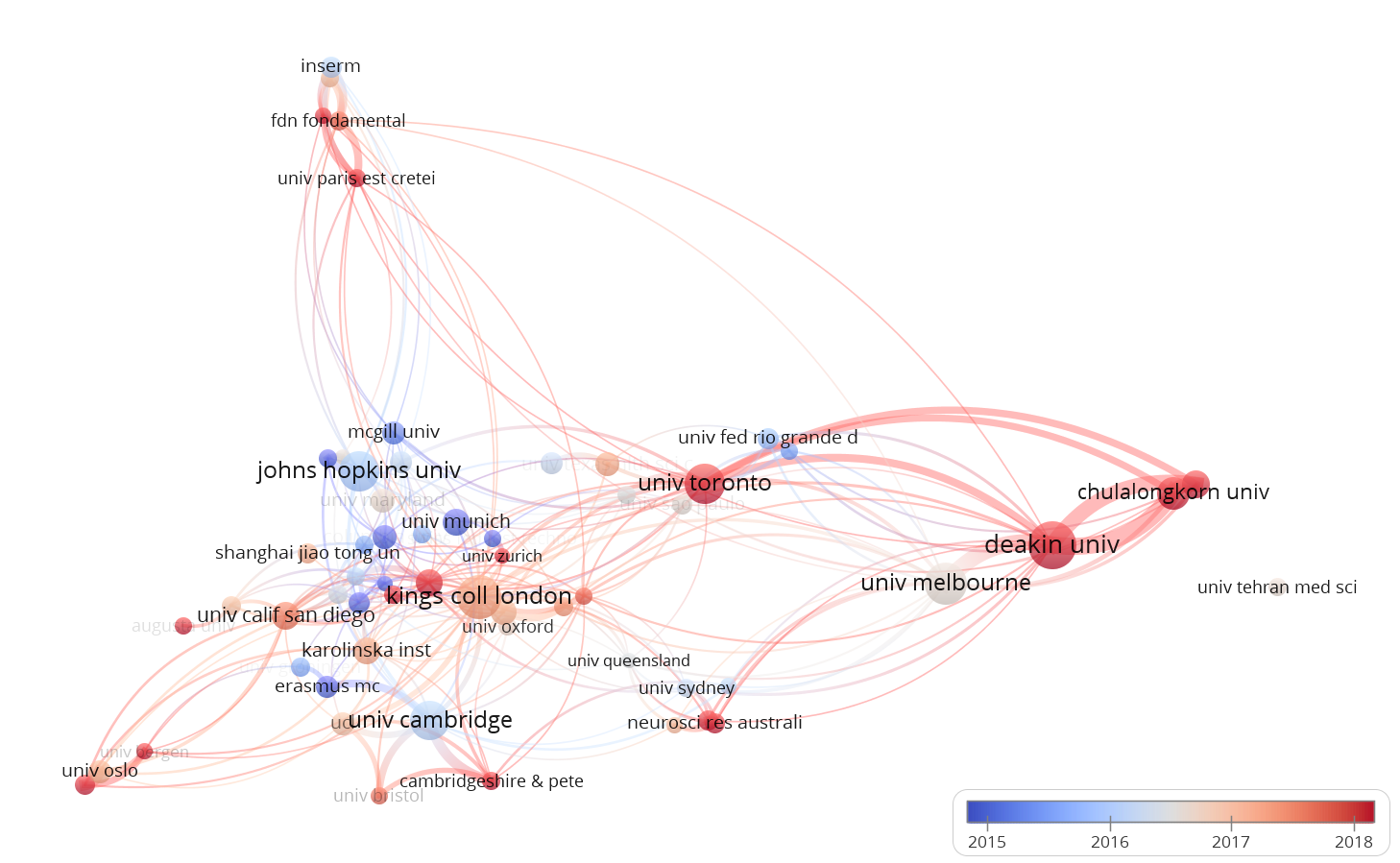


**Figure S2. Overlay visualization map of worldwide institutions institution co-authorship analysis on schizophrenia and inflammation research**


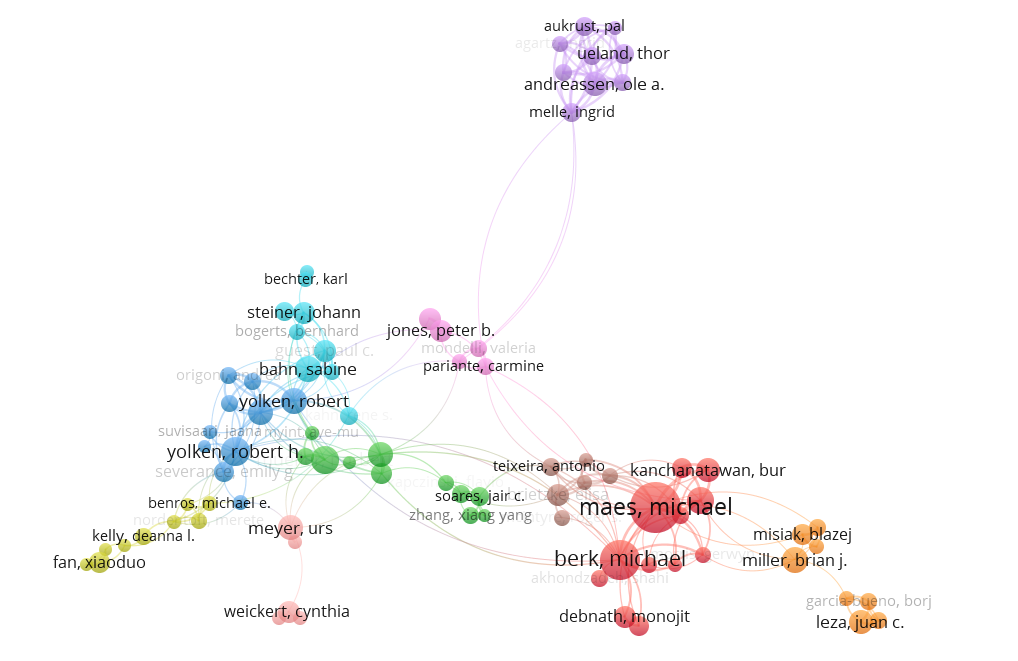


**Figure S3.** **Network visualization map of author co-authorship analysis on schizophrenia and inflammation**


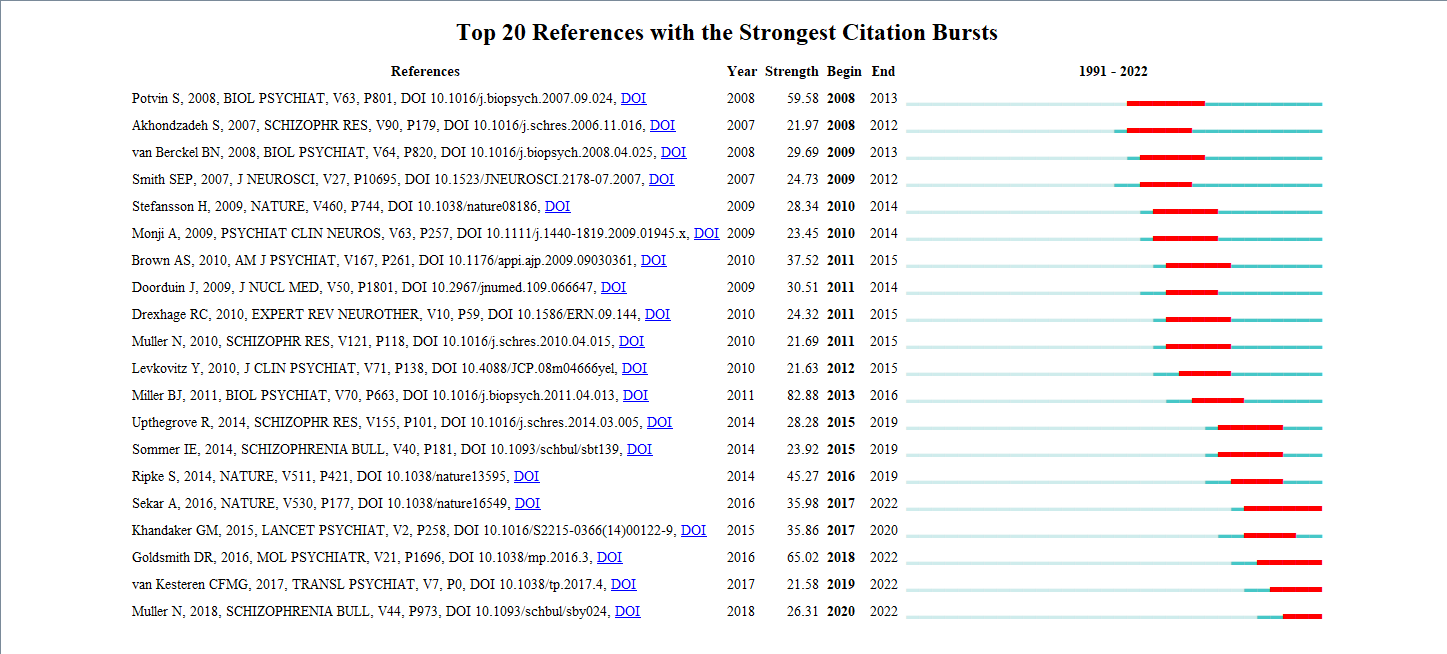


**Figure S4. Top 20 keywords with the strongest citation bursts on schizophrenia and inflammation**

**Table S1. The top 10 cited articles**

| **Title** | **Journal/Year** | **First Author** | **TC** | **TC/Y** |
| --- | --- | --- | --- | --- |
| Meta-analysis of cytokine alterations in schizophrenia: clinical status and antipsychotic effects | BIOL PSYCHIAT/2011 | MILLER BJ | 1,031 | 85.9 |
| Inflammatory cytokine alterations in schizophrenia: a systematic quantitative review | BIOL PSYCHIAT/2008 | POTVIN S | 704 | 46.9 |
| A meta-analysis of blood cytokine network alterations in psychiatric patients: comparisons between schizophrenia, bipolar disorder, and depression | MOL PSYCHIATR/2016 | GOLDSMITH DR | 638 | 91.1 |
| The time of prenatal immune challenge determines the specificity of inflammation-mediated brain and behavioral pathology | J NEUROSCI/2006 | MEYER U | 545 | 32.0 |
| The cyclooxygenase-2 inhibitor celecoxib has therapeutic effects in major depression: results of a double-blind, randomized, placebo controlled, add-on pilot study to reboxetine | MOL PSYCHIATR/2006 | MULLER N | 539 | 31.7 |
| Detection and interpretation of shared genetic influences on 42 human traits | NAT GENET/2016 | PICKRELL JK | 529 | 75.5 |
| Clinical use of current polygenic risk scores may exacerbate health disparities | NAT GENET/2019 | MARTIN AR | 509 | 127.2 |
| Increased serum IL-6 and IL-1 receptor antagonist concentrations in major depression and treatment resistant depression | CYTOKINE/1997 | MAES M | 505 | 19.4 |
| Immune involvement in schizophrenia and autism: etiology, pathology and animal models | BEHAV BRAIN RES/2009 | PATTERSON PH | 494 | 35.2 |
| Aspirin: a review of its neurobiological properties and therapeutic potential for mental illness | BMC MED/2013 | BERK M | 443 | 44.3 |
| Notes: TC: Total Citations; TC/Y: Average per Year Total Citations; | | | | |
